# Efficient Removal of Cr(VI) from Water by Carbon-Based Composites Derived from Oil-Based Drill Cuttings

**DOI:** 10.3390/ma18235295

**Published:** 2025-11-24

**Authors:** Yongkui Huang, Linzhi Li, Jiangrong Qiao, Xiaochuan Wu, Ye Zhang, Bo Bai, Liu Yang, Shubiao Pan, Qi Feng, Li Liu

**Affiliations:** 1 National and Local Joint Engineering Research Center of Shale Gas Exploration and Development, Key Laboratory of Shale Gas Exploration, Ministry of Natural Resources, Chongqing Institute of Geology and Mineral Resources, Chongqing 401120, China; xiaohuang2118@163.com (Y.H.); lilinzhi2025uk@outlook.com (L.L.); hsiaochuanwu@hotmail.com (X.W.); zhangye_sg@vip.163.com (Y.Z.); willowred@163.com (L.Y.); pan_shubiao@163.com (S.P.); 2 State Key Laboratory of Coal Mine Disaster Dynamics and Control, School of Resources and Safety Engineering, Chongqing University, Chongqing 400044, China; qjr20202020@163.com

**Keywords:** oil-based drill cuttings, carbon-based composites, Cr(VI) removal, adsorption performance

## Abstract

Oil-based drill cuttings (OBDCs) have received extensive attention for their environmental impacts and safe disposal. In this study, a high-performance sorbent material was prepared via the chelation and carbonization reaction for the high-value resource utilization of OBDCs. The coordination interaction between the organic acids and metal ions acted as a carbon source. Then, the metal elements were encapsulated into the formed carbon matrix. The results showed that the obtained composites exhibited abundant honeycomb-like pore structures. The addition of citric acid facilitated the formation of graphitized carbon materials and increased the specific surface area and surface functional groups. Impressively, the composites exhibited excellent adsorption performance for Cr(VI), with a maximum adsorption capacity of 56.84 mg/g. The adsorption process followed the pseudo-second-order kinetic model, and was mainly dominated by chemical adsorption. The excellent adsorption properties were attributed to the unique properties of the prepared carbon-based composites, including their large surface area, numerous pore structures, and abundant surface functional groups. This study provides new ideas related to the resource utilization of hazardous organic waste.

## 1. Introduction

Shale gas, as a clean, efficient and unconventional fossil energy source, plays an important role in alleviating the pressure of energy supply and demand and achieving the strategic goals of carbon neutrality [1,2,3,4]. Long-distance horizontal drilling with extensive use of oil-based drilling fluids is one of the key technologies for improving the recovery rate of shale gas. Nevertheless, this technology inevitably generates a large amount of oil-based drill cuttings (OBDCs) [1,5,6]. These cuttings contain significant amounts of toxic substances, such as petroleum hydrocarbons, heavy metals, and polymeric organic pollutants [3,7,8]. In addition, due to their high biological toxicity, OBDCs have poor degradability in natural environments and can be absorbed by crops and other vegetation, entering the food chain and ultimately affecting human health [2,9,10]. Therefore, the effective treatment of OBDCs remains a critical challenge for the sustainable development of the shale gas industry [3,11,12]. In recent years, various techniques have been proposed to achieve the harmless treatment of OBDCs, such as centrifugation, reinjection, landfilling, incineration, microbial treatment, and solidification technologies [11,13,14,15]. However, these methods are often plagued by low resource utilization efficiency and the inherent risk of secondary pollution [10,16,17]. To achieve successful resource utilization of oil-based drill cuttings, significant progress has been made in the development of related technologies, such as surfactant-enhanced washing, supercritical fluid extraction, and microwave pyrolysis [18,19,20,21]. These technologies can recover most of the oil phase components, but the residual solid still contains polymeric organic pollutants and heavy metals, and is still regarded as hazardous waste [22,23,24]. Therefore, the comprehensive valorization of oil-based drill cuttings has become an important research direction.

The resource utilization of oil-based drill cuttings shows a trend of expanding from low-value-added applications to high-value environmental remediation fields [25,26,27]. In recent years, researchers have focused on environmental remediation using OBDC residues, aiming to achieve the sustainable development goal of “treating waste with waste” [7,23,28]. Due to the presence of transition metals and alkali metal minerals in the residues, they have the potential to be used as adsorbents, reducing agents, and catalysts in environmental remediation [16,29,30]. For instance, OBDCs can function as a reducing agent due to their reductive sulfur and phosphorus content [4]. Yang et al. confirmed that secondary pyrolysis oil-based drill cutting ash can effectively oxidize heavy metal pollutants in water and soil by virtue of these reductive and precipitation characteristics [30]. Furthermore, OBDC contains numerous alkaline metal oxides and possesses a surface endowed with functional groups with tunable hydrophilic/hydrophobic properties, which provides an opportunity to improve their adsorption performance and selectivity for wastewater treatment. Zhao et al. and Yang et al. have demonstrated that the adsorption performance of post-pyrolysis OBDC is significantly enhanced after oxidative modification [31,32]. However, inhibiting the ecological risk of heavy metals leaching from OBDC solids remains challenging [1,33]. Therefore, it is urgent to develop effective and cost-effective synthesis strategies to convert organic pollutants and metal elements from OBDC into high-performance functional materials.

In this study, a fast and scalable approach has been developed to prepare a high-performance sorbent material by utilizing the chelation effect between organic acids and OBDCs combined with carbonization reaction for high-value resource utilization of OBDCs. The coordination interaction between the organic acids and metal ions in OBDCs can serve as ionic cross-linkers and can act as a carbon source. Then, the metal elements in oil-based cuttings are encapsulated into the formed carbon matrix to achieve solidification after a calcination process. Finally, the removal of hexavalent chromium (Cr(VI)) was used as a model reaction to explore the adsorption performance of the obtained carbon-based composites for the remediation of heavy metal pollution. This paper provides a theoretical basis and technical references for “treating waste with waste” for high-value applications in environmental remediation.

## 2. Materials and Methods

### 2.1. Preparation Method

The oil-based drill cuttings were collected from the Shale Gas Field in Yuxi District, Chongqing, China. First, the oil was removed via centrifugal separation. The oil content of the resulting solid product was about 0.58%. After the product was dried at 105 °C, a grayish-brown residue was obtained and served as the precursor for the study.

The carbonization device was composed of a quartz tube and a horizontal pyrolysis furnace, with electric resistance wires as the heating element. To eliminate the impact of the solution’s acidity, sodium citrate was selected as the source of citric acid. The specific preparation steps were as follows: 5 g of the precursor and 3 g of sodium citrate were added to 20 mL of water and stirred for 2 h. After drying at 80 °C overnight, the mixture was ground uniformly. Subsequently, the obtained mixture was placed into the quartz tube of the tube furnace and calcined under a nitrogen atmosphere for a duration of 120 min. After cooling to room temperature, the obtained product was collected and washed several times with distilled water. Moreover, the effect of carbonization temperature on the structure and adsorption performance of carbon-based composites was studied. The obtained products with different carbonization temperatures (500, 600, 700, and 800 °C) were labeled as DCS 1, DCS 2, DCS 3, and DCS 4, respectively. In addition, a material obtained via the same processes without the addition of sodium citrate was used as a control and labeled as DC.

### 2.2. Cr(VI) Adsorption Experiment

Batch adsorption experiments were performed to verify the adsorption performance of the carbon-based composites. Typically, a Cr(VI) solution (50 mg/L) was prepared by dissolving potassium dichromate (K_2_Cr_2_O_7_, analytical grade) in distilled water. Next, 0.05 g of the carbon-based composite sample was added to 100 mL of Cr(VI) solution. The flask was sealed with a stopper and placed in a constant-temperature shaking incubator (Shanghai bluepard instruments CO., LTD., Shanghai, China) with a shaking speed of 120 rpm for 24 h. After adsorption, the material and solution were separated using a 0.45 μm filter membrane. The Cr(VI) concentration was determined via the 1,5-diphenylcarbazide analytical method at 540 nm using a UV1102 II ultraviolet spectrophotometer(Beijing Puxi General Instrument Co., Ltd., Beijing, China) [34,35].

The adsorption kinetics experiments were conducted at 25 °C, with an adsorbent dosage of 0.5 g/L and an initial Cr(VI) concentration of 50 mg/L. The concentrations of Cr(VI) were monitored regularly throughout the process. The pseudo-first-order kinetic model and the pseudo-second-order kinetic model were used to explore the kinetic characteristics of Cr(VI) adsorption by the adsorbent.

The adsorption capacity (*q_t_*) and the kinetic parameters was calculated using the following formula:

(1) $$q_{t}=\frac{(c_{0}-c_{t})\times v}{m}$$

(2) $$q_{t}=q_{e}(1-e^{-k_{2}\times t})$$

(3) $$\frac{t}{q_{t}}=\frac{t}{q_{e}}+\frac{1}{k_{2}\times q_{e}^{2}}$$

where *c*_0_ and *c_t_* represent the initial concentration and at time *t* of Cr (VI) (mg/L), respectively, *v* is the volume of the solution (L), and *m* is the mass of the adsorbent (g). *q_e_* and *q_t_* represent the equilibrium adsorption capacity and the adsorption capacity at time *t* (mg/g). *k*_1_ and *k*_2_ is the pseudo-first-order (min^−1^) and the pseudo-second-order kinetic rate constant (g·mg^−1^·min^−1^).

### 2.3. Characterization and Analysis Methods

Elemental analysis of the samples was performed using CHNS analysis on a Unicube elemental analyzer (Elementar Trading (shanghai) Co., Ltd., Shanghai, China). The crystal phase of the samples was characterized by powder X-ray diffraction (XRD, PANalytical X’Pert, Osaka, Japan) equipped with Cu Kα radiation in the range of 6° to 60°. The surface functional groups were analyzed using a VERTEX 70 Fourier transform infrared (FTIR) spectrometer (Bruker Corporation, Karlsruhe, Germany) with the KBr pellet method in the range of 4000 to 400 cm^−1^. Raman spectra of the samples were obtained using a LabRAM HR Evolution Raman spectroscope (HORIBA, Palaiseau, France) with a 514 nm laser source. The mineral components were investigated using an X-ray fluorescence (XRF) spectrometer equipped with a Rh standard tube as the excitation source (ARL Perform’X, Thermo Fisher Scientific, Waltham, MA, USA). The specific surface area and pore size were analyzed by N_2_ adsorption–desorption measurements (Micromeritics ASAP 2020, Norcross, GA, USA) at 77 K. The samples were degassed under a vacuum at 100 °C for 10 h prior to the experiments. The morphology and elemental composition were characterized using Scanning Electron Microscopy (SEM) (JSM-7800F microscope, Tokyo, Japan) at an acceleration voltage of 15 kV. The surface elements were identified by an X-ray photoelectron spectrometer (XPS, ESCALAB250Xi, Thermo Fisher Scientific) with monochromatic Al-Kα radiation, and the binding energies were calibrated by C 1s at 284.8 eV.

## 3. Results and Discussion

### 3.1. Structural Characterization of Carbon-Based Composites

The XRF measurement was performed to determine the inorganic mineral composition of the synthesized materials. As illustrated in Table 1, the mineral compositions of the different carbon-based composites were largely identical. Notably, the primary constituents were identified as SiO_2_, Al_2_O_3_, CaO, Ba, Fe_2_O_3_, and Na_2_O [9,11,28]. This elemental profile is fundamentally consistent with that of the raw oil-based drill cuttings precursor, with only slight changes observed in the relative proportions of the components after the synthesis process.

Elemental analysis was performed to identify the relative content of carbon (C) and hydrogen (H) within the carbon-based composites. As shown in Table 2, all the synthesized composites contained C and H. Notably, the elemental contents of C and H in DC were significantly lower than those in the carbon-based composites. This indicates that the citric acid had undergone a carbonization reaction and had been converted into structural carbon material. Furthermore, the C and H content in the samples markedly decreased with increasing carbonization temperature. Concurrently, the atomic ratio of C/H also progressively decreased with rising temperature. This was mainly attributed to the fact that organic substances and citric acid were transformed into carbon structures. These results confirm that the organic matter was effectively converted into a carbonaceous material.

**Figure 1:**
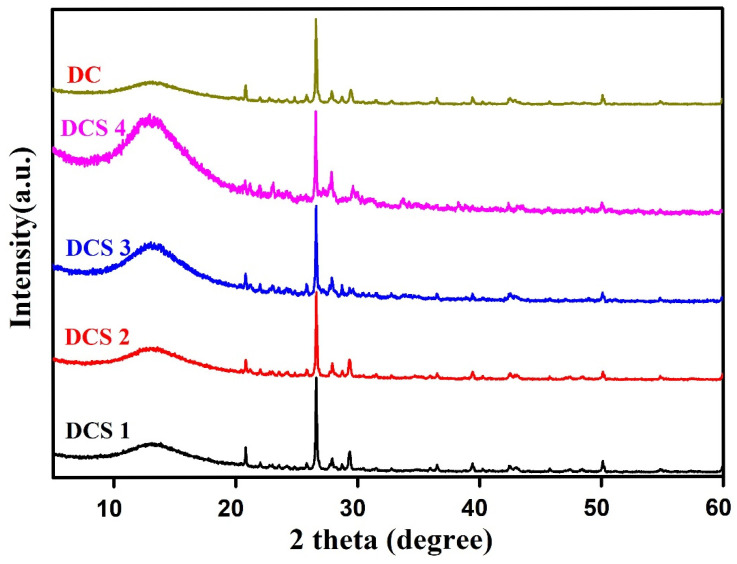
XRD analysis of samples.

The phase composition of the synthesized carbon-based composites was investigated using XRD. As shown in Figure 1, the main phase composition was consistent across the materials prepared at different temperatures, such as quartz, calcite, potassium feldspar, and barium sulfate. A relatively strong diffraction peak was observed at 2θ = 26.6°, corresponding to the characteristic peak of quartz (PDF:99-0088) [10,29,32]. Moreover, the diffraction intensity progressively increased with increasing reaction temperature, suggesting that higher temperatures promoted the growth and crystallinity of this phase. Furthermore, as the reaction temperature increased, the diffraction peak initially located at 29.3° gradually shifted to 29.6°, which may have been due to the partial decomposition of BaCO_3_ and CaCO_3_ in the residues [10,28]. No other significant changes were observed in the other crystalline phases. These results demonstrate that the carbon-based composites retained the original mineral composition of the precursor material.

**Figure 2:**
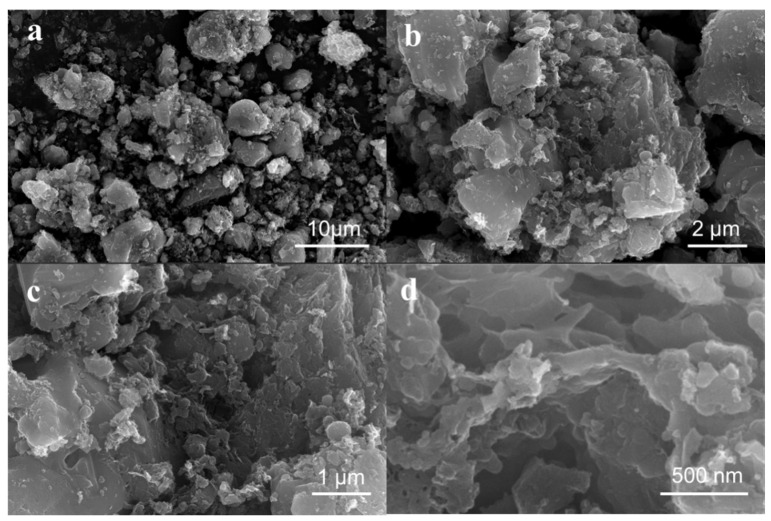
SEM analysis of DCS 3 at different magnifications (**a**–**d**).

Figure 2 shows the morphologies of the synthesized carbon materials. As observed in Figure 2a, DCS 3 is composed of irregularly shaped particles with sizes ranging from several hundred nanometers to approximately 15 µm. Figure 2b,c reveal that the particle surfaces possess an abundant, honeycomb-like porous architecture with many pores and grooves, which are beneficial for providing more adsorption sites and a larger specific surface area [13,17,36]. The high-resolution SEM image (Figure 2d) shows that a thin carbon layer formed on the surfaces of the materials. Additionally, the carbon layer presented a large number of irregular, pore-like structures.

**Figure 3:**
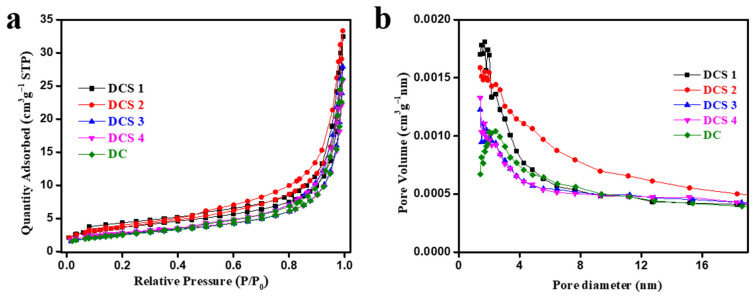
N_2_ adsorption–desorption isotherms (**a**) and pore size distribution curves (**b**) of the samples.

The specific surface area and pore structures of the materials were further analyzed using N_2_ adsorption–desorption isotherms. As shown in Figure 3a, all the samples presented a type IV isotherm with an H3 hysteresis loop, indicating the presence of typical mesoporous and macroporous structures [36]. Figure 3b shows that the pore sizes of the samples were mainly distributed below 8 nm. As listed in Table 3, the average pore sizes of DCS 1, DCS 2, DCS 3, and DCS 4 were 16.44, 15.21, 18.32, and 17.31 nm, respectively, indicating that the increase in carbonization temperature promoted the carbonization of organic matter and unblocked the pore structures. In addition, the addition of citric acid may have increased the specific surface area. However, the specific surface area decreased with the increase in the carbonization temperature, which could be attributed to the transformation of the citric acid. Therefore, carbonization temperature plays a crucial role in the formation of micro/mesopores.

**Figure 4:**
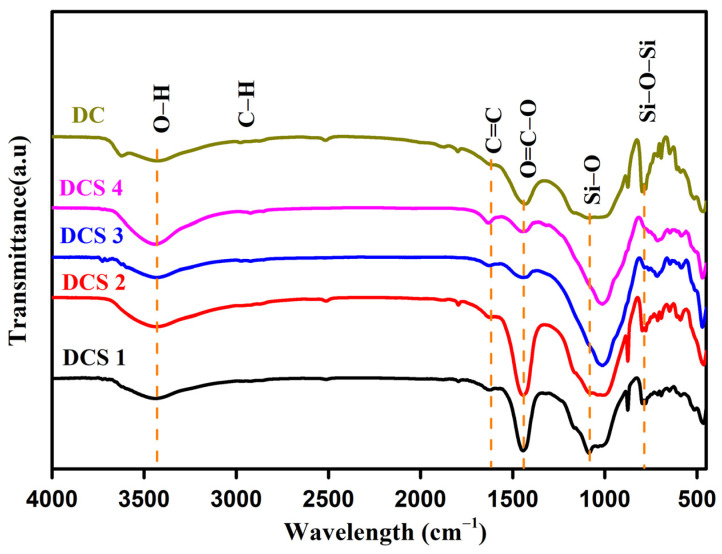
FT-IR spectra analysis of samples.

The surface functional groups of the samples were analyzed by FT-IR [37]. As shown in Figure 4, the peak at 3450 cm^−1^ was attributed to the stretching vibration of –OH, and the peak at ~2850 cm^−1^ corresponded to the stretching vibration of C–H. The peak at 1632 cm^−1^ was assigned to the stretching vibration of C=C in pyridine structures, and the characteristic peak near 1443 cm^−1^ could be attributed to the vibrational absorption of aromatic skeletons [38]. Furthermore, the characteristic peak near 1443 cm^−1^ became very weak with the increase in the reaction temperature, while the peak at 1632 cm^−1^ became stronger, indicating that the aromatic skeleton had begun to transform into graphite layers or carbon black structures. The characteristic peak near 1100 cm^−1^ was attributed to the vibration of Si−O, and the peak between 780 and 790 cm^−1^ corresponded to the symmetric vibration of Si−O−Si, indicating the presence of silicates [30,39]. Therefore, the surface of the obtained composites was found to be decorated with various functional groups, which are beneficial to the adsorption performance.

**Figure 5:**
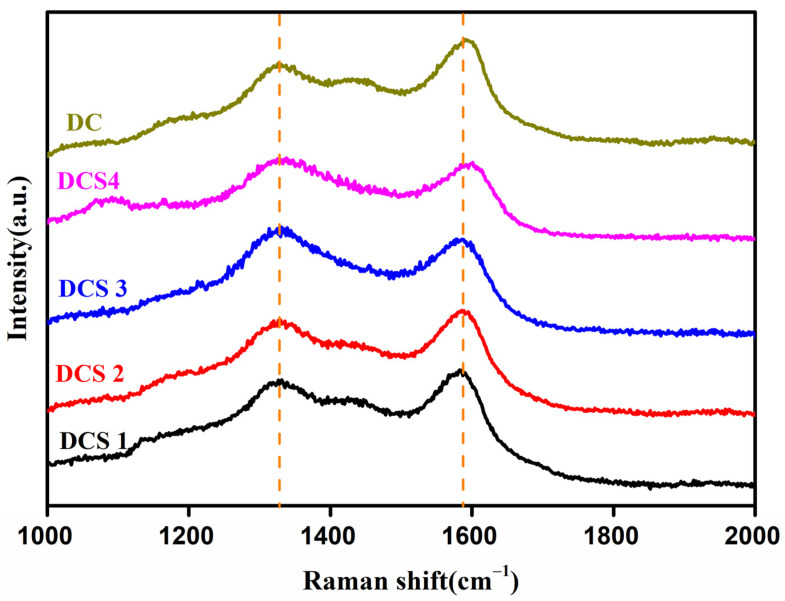
Raman spectra analysis of samples.

Figure 5 displays the Raman spectroscopy of the obtained carbon-based composites. The obtained composites have two obvious characteristic peaks at 1323 cm^−1^ and 1586 cm^−1^, corresponding to the D band and G band of carbon materials, respectively [36,40]. The calculated I_D_/I_G_ values of DCS 1, DCS 2, DCS 3, and DCS 4 are 0.91, 0.93, 0.93, and 1.06, respectively. In contrast, the I_D_/I_G_ value of the DC sample is 0.83, indicating that the graphitization degree of its carbon materials is relatively low. This illustrated that a higher carbonization temperature was beneficial for improving the graphitization degree of the carbon materials. This indicates that the addition of citric acid was beneficial to the formation of graphitized carbon materials, leading to the synthesis of a higher-performance carbon material.

**Figure 6:**
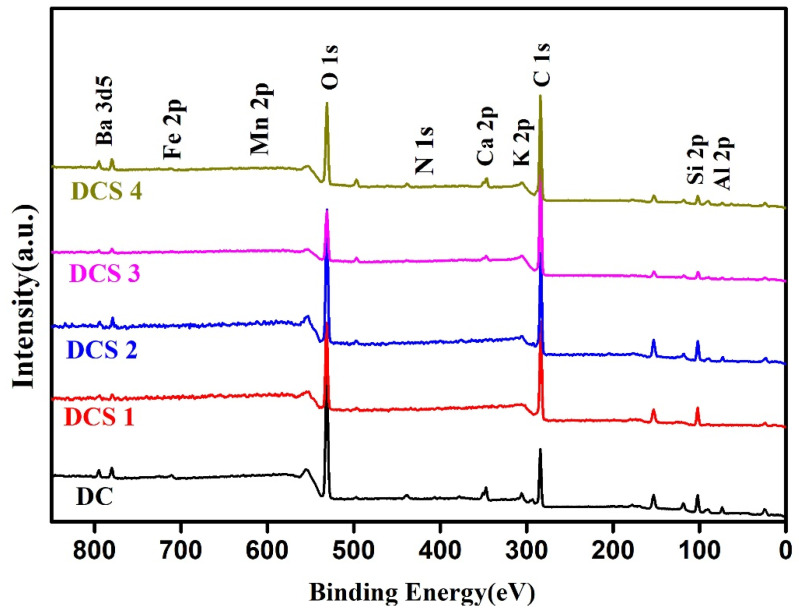
XPS spectra of samples.

XPS was used to further investigate the surface structure of the obtained composites. As shown in Figure 6, all the samples contained C, O, Si, Al, Ba, and Ca elements. Notably, the atomic percentages of carbon (C) in DC, DCS 1, DCS 2, DCS 3, and DCS 4 were 35.55, 53.35, 65.2, 76.05, and 64.47. It can be concluded that a large amount of carbon materials mainly derived from the carbonization of citric acid formed on the surface of the obtained composites. Furthermore, trace amounts of Fe and Mn were detected in the DC sample. In contrast, no obvious signals were observed in the obtained composites. This can be attributed to the fact that the thick carbon layer that formed on the surface of the sample effectively encapsulated the underlying mineral elements. This is not only beneficial for improving the adsorption performance but is also conducive to the immobilization of metal elements.

### 3.2. Adsorption Performance

Based on the above results, the synthesized carbon-based composites feature a typical mesoporous architecture and are rich in functional groups. Therefore, the removal of Cr(VI) was used as a model reaction to explore the application potential of the obtained composites in pollutant remediation.

**Figure 7:**
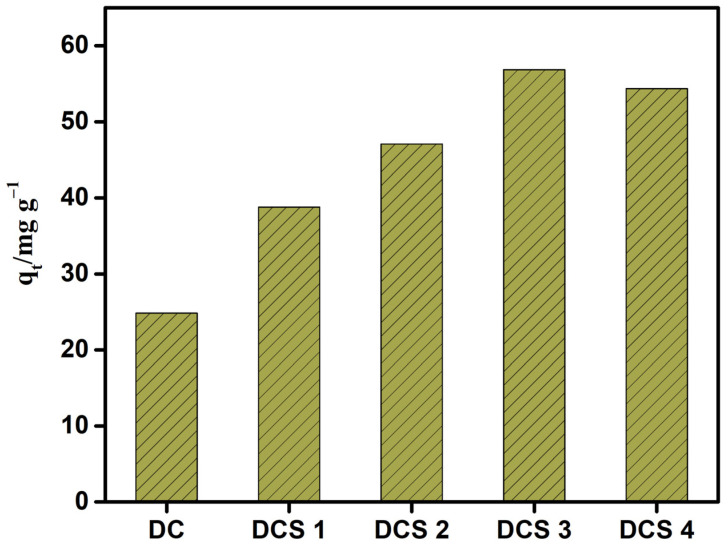
Trends in the adsorption performance of the obtained materials for Cr(VI).

As clearly shown in Figure 7, the DC exhibited a poor adsorption performance for Cr(VI) with an equilibrium adsorption capacity of 24.83 mg/g, which may have been due to the low amount of formed carbon materials. As expected, a significant improvement in adsorption capacity was observed for the composites prepared with citric acid. As the carbonization temperature increased from 500 °C to 700 °C, the adsorption capacity increased from 38.79 to 56.84 mg/g. However, a further increase in carbonization temperature slightly decreased the adsorption capacity. This may have been due to the high carbonization temperature, which caused a significant loss of carbon materials. It can be concluded that the carbonization temperature has a significant impact on the adsorption performance of the formed carbon-based composites, and the carbon-based composite prepared at 700 °C exhibited the best adsorption effect.

**Figure 8:**
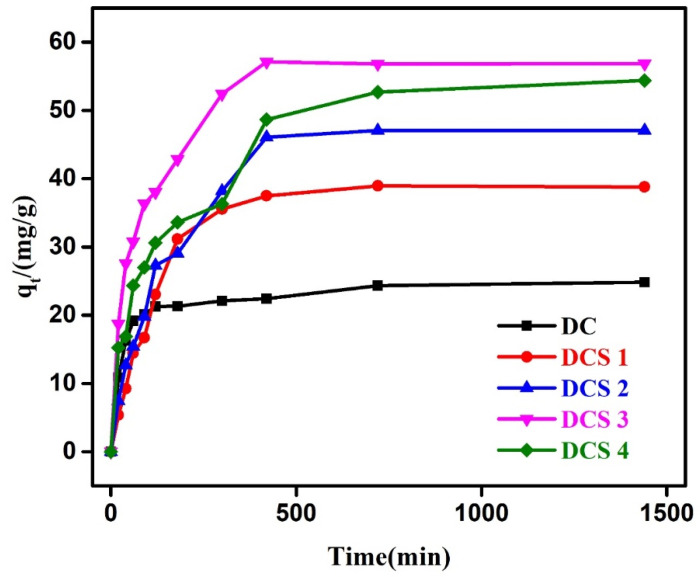
Adsorption kinetics of the samples.

Figure 8 shows the adsorption kinetic curves of Cr(VI) on the carbon-based composites. The adsorption performance of the carbon-based composites gradually increased with the extension of time, and an adsorption equilibrium could be reached at approximately 420 min. The adsorption performance of the carbon-based composites increased slightly with the increase in adsorption time. This may have been because the abundant porous structure of the carbon-based composites facilitates the transport and diffusion of Cr(VI) from the material’s surface to the interior. In addition, the adsorption capacity of the DCS 3 sample slightly decreased with the increase in adsorption time. This may have been due to the large potential energy difference between Cr(VI) adsorbed on the surface of DCS 3 and Cr(VI) in the solution, promoting the transfer of Cr(VI).

To further evaluate the adsorption performance of the synthesized composites, a comparison was made with other reported adsorbents. As shown in Table 4, the synthesized carbon-based composites exhibited a significantly higher adsorption performance than most of the current mainstream adsorbents. Therefore, the synthesized composites have promising application prospects in Cr(VI) wastewater treatment, serving as environmentally friendly and potentially cost-effective adsorbent materials.

### 3.3. Adsorption Kinetics

**Figure 9:**
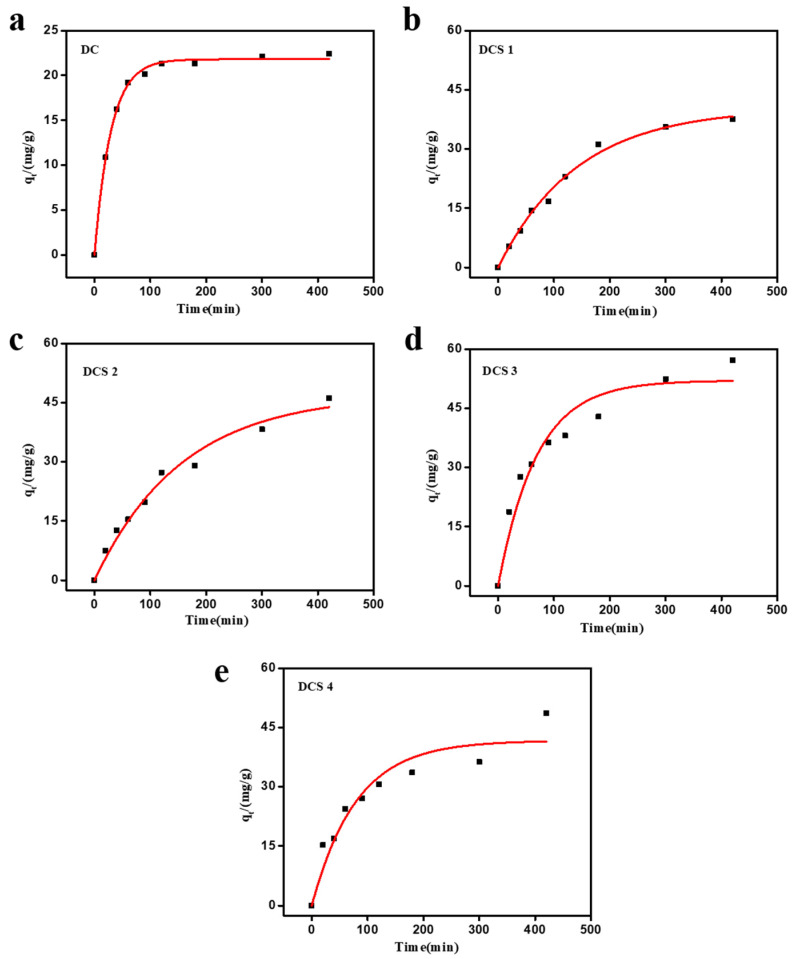
Pseudo-first-order kinetic fitting curves of Cr(VI) adsorption by DC (**a**), DCS 1 (**b**), DCS 2 (**c**), DCS 3 (**d**),and DCS 4 (**e**).

**Figure 10:**
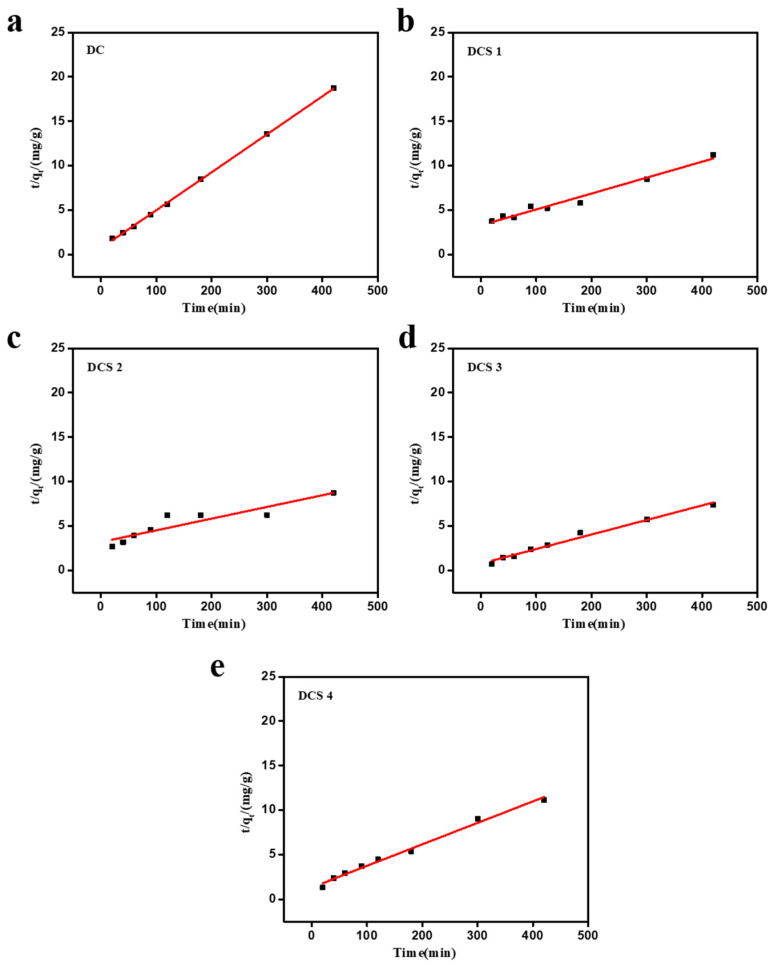
Pseudo-second-order kinetic fitting curves of Cr(VI) adsorption by DC (**a**), DCS 1 (**b**), DCS 2 (**c**), DCS 3 (**d**), and DCS 4 (**e**).

To reveal the adsorption behavior of the prepared carbon-based composites, pseudo-first-order and pseudo-second-order kinetic equations were utilized to understand the adsorption isotherms of the prepared composites [16,30,44,45]. As shown in Figure 9, the adsorption process had a relatively good correlation with the pseudo-first-order kinetic model. The correlation coefficients (R^2^) of DCS 1, DCS 2, DCS 3, and DCS 4 samples were 0.99, 0.98, 0.94, and 0.92, respectively. Moreover, their experimental equilibrium adsorption capacities were closer to the theoretical equilibrium adsorption capacities. In addition, the rate constant *k* of the DC sample was significantly higher than that of the prepared composites. This may suggest that the adsorption process of the DC sample is primarily dominated by an intraparticle diffusion mechanism [34,35]. This observation is consistent with previous studies [34,35] in which pristine biochar exhibited higher apparent rate constants due to diffusion-controlled uptake, whereas modified biochar showed adsorption behavior better fitted by the pseudo-second-order model, highlighting the dominant role of chemical interactions in pollutant removal. As shown in Figure 10a and Table 5, both the pseudo-first-order and pseudo-second-order models adequately described the adsorption process; however, the pseudo-second-order model yielded a more reliable prediction of the equilibrium adsorption capacity for most composites [46,47]. This result suggests that the adsorption of Cr(VI) by the prepared materials was predominantly governed by chemisorption [16,34,35]. The excellent adsorption properties are attributed to the unique properties of the prepared carbon-based composites, including their large surface area, numerous pore structures, and abundant surface functional groups.

## 4. Conclusions

In summary, novel carbon-based composites were successfully synthesized by utilizing the chelation reaction of oil-based cuttings and carbonization treatment for the removal of Cr(VI) in water. The carbon-based composites are basically consistent with the mineral composition of the raw oil-based cuttings. The composites exhibited irregular particle morphology with abundant honeycomb-like pore structures. The thin carbon layer with a large number of irregular porous structures was formed on the surface. Various functional groups were decorated on the surface of the obtained composites. The addition of citric acid is beneficial for the formation of graphitized carbon materials and can increase the specific surface area. The specific surface area decreases with the increase in the carbonization temperature. The composites exhibited excellent adsorption performance for Cr(VI), with a maximum adsorption capacity of 56.84 mg/g at a carbonization temperature of 700 °C. The adsorption process followed the pseudo-second-order kinetic model and was mainly dominated by chemical adsorption. The excellent adsorption properties are attributed to the unique properties of the prepared carbon-based composites, including their large surface area, numerous pore structures, and abundant surface functional groups. This work provides new ideas and approaches for the high-value valorization of oil-based drill cuttings.

## Figures and Tables

**Table 1:** Analysis of mineral composition of samples.

	Element	Unit	Sample Number				
			DC	DCS 1	DCS 2	DCS 3	DCS 4
Al_2_O_3_	Al	%	10.05	9.09	9.24	10.77	10.82
CaO	Ca	%	7.62	8.06	7.69	7.13	7.64
Fe_2_O_3_	Fe	%	2.60	2.59	2.51	2.67	2.43
K_2_O	K	%	1.47	1.44	1.32	1.55	1.47
MgO	Mg	%	0.85	0.78	0.72	0.85	0.79
Na_2_O	Na	%	2.45	3.24	5.66	8.14	8.07
SiO_2_	Si	%	55.30	50.88	49.03	49.41	47.76
Ba	Ba	%	3.53	3.17	3.24	3.53	3.56
Mn	Mn	%	0.22	0.22	0.21	0.23	0.20
Ti	Ti	%	0.98	0.89	0.90	0.97	0.98

**Table 2:** Analysis of C and H content in the samples.

Sample Number	C [%]	H [%]	C/H
DCS 1	5.62	0.445	12.65
DCS 2	4.97	0.447	11.12
DCS 3	3.18	0.282	11.28
DCS 4	2.23	0.212	10.49
DC	1.94	0.16	12.13

**Table 3:** Analysis of the specific surface area, pore volume, and average pore size of the samples.

Sample Name	Specific Surface Area (m^2^/g)	Pore Volume (cm^3^/g)	Average Pore Size (nm)
DC	9.06	0.040	17.08
DCS 1	12.82	0.049	16.44
DCS 2	13.26	0.051	15.21
DCS 3	9.27	0.043	18.32
DCS 4	9.64	0.040	17.31

**Table 4:** Performance comparison of related materials.

Raw Material	Synthesis Temperature	q_m_/(mg/g)	Reference
Bi_5_O_7_I/ZnAlBi-CLDHs-1/10	-	18.73	[35]
Bismuth impregnated biochar	600	12.20	[41]
Activated carbon	30	43.45	[42]
Hydrochar by biomass with ZnCl_2_	200	14.00	[43]
Fe@MBC	600	25.46	[34]
Carbon-Modified Oil-Based Cuttings	700	56.84	This Study

**Table 5:** Kinetic fitting parameters of Cr(VI) adsorption by prepared materials.

Adsorbent Material	Pseudo-First-Order Kinetics		Pseudo-Second-Order Kinetics	
	*k*_1_ (min^−1^)	R^2^	*k*_2_ (g·mg^−1^·min^−1^)	R^2^
DC	0.03399	1.00	0.002592	1.00
DCS 1	0.00703	0.99	0.0001	0.97
DCS 2	0.00638	0.98	5.46 × 10^−5^	0.85
DCS 3	0.01452	0.94	0.000355	0.99
DCS 4	0.01255	0.92	0.000443	0.99

## Data Availability

The original contributions presented in this study are included in the article. Further inquiries can be directed to the corresponding authors.
